# Hearing loss and depressive symptoms in older Chinese: whether social isolation plays a role

**DOI:** 10.1186/s12877-022-03311-0

**Published:** 2022-07-26

**Authors:** Hao Huang, Jiao Wang, Chao Qiang Jiang, Feng Zhu, Ya Li Jin, Tong Zhu, Wei Sen Zhang, Lin Xu

**Affiliations:** 1 School of Public Health, Sun Yat-Sen University, No. 74 Zhongshan 2ndRoad, Guangzhou, Guangdong Province China; 2 Guangzhou Twelfth People’s Hospital, Guangzhou, 510620 China; 3 School of Public Health, the University of Hong Kong, Hong Kong, China

**Keywords:** Hearing loss, Depressive symptoms, Social isolation

## Abstract

**Background:**

Existing evidence links hearing loss to depressive symptoms, with the extent of association and underlying mechanisms remaining inconclusive. We conducted a cross-sectional study to examine the association of hearing loss with depressive symptoms and explored whether social isolation mediated the association.

**Methods:**

Eight thousand nine hundred sixty-two participants from Guangzhou Biobank Cohort Study were included. Data on self-reported hearing status, the 15-item Geriatric Depression Scale (GDS-15), social isolation and potential confounders were collected by face-to-face interview.

**Results:**

The mean (standard deviation) age of participants was 60.2 (7.8) years. The prevalence of poor and fair hearing was 6.8% and 60.8%, respectively. After adjusting for age, sex, household income, education, occupation, smoking, alcohol use, self-rated health, comorbidities, compared with participants who had normal hearing, those with poor hearing (β = 0.74, 95% confidence interval (CI) 0.54, 0.94) and fair hearing (β = 0.59, 95% CI 0.48, 0.69) had higher scores of GDS-15. After similar adjustment, those with poor hearing (odds ratio (OR) = 2.13, 95% CI 1.65, 2.74) or fair hearing (OR = 1.68, 95% CI 1.43, 1.99) also showed higher odds of depressive symptoms. The association of poor and fair hearing with depressive symptoms attenuated slightly but not substantially after additionally adjusting for social isolation. In the mediation analysis, the adjusted proportion of the association mediated through social isolation was 9% (95% CI: 6%, 22%).

**Conclusion:**

Poor hearing was associated with a higher risk of depressive symptoms, which was only partly mediated by social isolation. Further investigation of the underlying mechanisms is warranted.

**Supplementary Information:**

The online version contains supplementary material available at 10.1186/s12877-022-03311-0.

## Background

Depressive symptoms is the most common mental disorder in older people [1] and a leading cause of disability worldwide [2]. In China, the prevalence of depressive symptoms in people aged over 60 years was 22.7% [3]. Such high prevalence highlights the need for more effective preventive efforts.

Hearing loss is another common health problem in older people, leading to communication difficulties. In 2015, more than half of adults in China aged over 60 years were affected by hearing loss [4]. Hearing loss has been associated with many adverse health outcomes, such as social loneliness, disability, frailty and increasing mortality [5–7].

Moreover, existing evidence also links hearing loss to depression [8–14], although the results were not completely consistent (i.e., three studies showed no association [6, 15, 16]). An extensive systematic review and meta-analysis of 35 studies showed that hearing loss was associated with 47% higher risk of depression in older adults, albeit a high degree of heterogeneity (I^2^ = 83.26%) [17].

Hearing loss may lead to communication difficulties, and subsequent feelings of being emotionally isolated and social isolated [6, 18–20]. It has been shown that social isolation is one of risk factors for depression [21–24], and the association between hearing loss and depressive symptoms was attenuated after adjustment for social engagement [25]. Therefore, social isolation may mediate the association between hearing loss and depression. However, whether and the extent to which social isolation mediates the association of hearing loss and depressive symptoms remain to be examined.

Hence, we examined the association between hearing loss and depressive symptoms using data from the Guangzhou Biobank Cohort Study (GBCS), a well-designed on-going population-based cohort study of older Chinese [26]. We also explored the possible mediating effect of social isolation on the association between hearing loss and depressive symptoms.

## Methods

### Study sample

GBCS is a three-way collaboration among the Guangzhou Twelfth People’s Hospital in Guangzhou and the University of Hong Kong in Hong Kong, China, and the University of Birmingham in UK. Details of the GBCS have been reported elsewhere [26]. Briefly, all participants were recruited from the Guangzhou Health and Happiness Association for the Respectable Elders (GHHARE), a large social and welfare organization. Guangzhou permanent residents aged 50 + years were eligible to participate and every member paid a monthly nominal fee of 4 RMB (about 0.57 USD). The GHHARE included about 7% of permanent Guangzhou residents in this age group, with branches over all districts of Guangzhou. All data were collected at Molecular Epidemiology Research Center in the Guangzhou Twelfth People’s Hospital during 2006–2008. Face-to-face interviews were conducted by full-time trained nurses. Physical examinations and laboratory assays were conducted by physicians and laboratory technicians in the hospital. The study was approved by the Guangzhou Medical Ethics Committee of the Chinese Medical Association. All participants provided written informed consent before participation.

### Exposure

Information on hearing impairment was assessed by self-reports at baseline [27]. Participants were asked “how is your hearing without a hearing aid” and the responses included “excellent, good, fair, poor, or unable to hear”. Participants who reported “excellent” or “good” hearing were categorized as good hearing, and “poor” or “unable to hear” as poor hearing, and thus three groups (good, fair, and poor hearing) were used in data analysis.

### Outcomes

Depressive symptoms were assessed by a previously validated Chinese version of the 15-item geriatric depression scale (GDS-15) [28]. The GDS-15 score ranges from 0 to 15, with higher scores indicating more depressive symptoms [29]. The presence of depressive symptoms was defined by a GDS-15 score equals to or higher than 5 [30, 31].

### Potential mediator

The potential mediator was social isolation. We used the Berkman-Syme Social Network Index (SNI) with appropriate revision [32, 33], which has been validated in Chinese population [34]: (1) Face-to-face contacts with co-inhabitants (including people who lived together); (2) Face-to-face contacts with co-inhabitants (excluding people who lived together); (3) Non-face-to-face contacts (by telephone/mail); or (4) club/organization contacts. We additionally included mail as another way of non-face-to-face contact besides telephone [33] to the Berkman-Syme Social Network Index (SNI), since both telephone and mail were common non-face-to-face contact ways in 2003, before smartphone, Internet, and social media had become popular. The four types of social isolation question and the scoring criteria for the composite social isolation score were shown in the Supplementary Table 1. A composite social isolation score was derived from the sum of 4 types of social isolation, with a score from 0 to 7. A higher score indicates more severe social isolation. The presence of social isolation was defined by a score of two or more.

### Potential confounders

Potential confounders adjusted in the multivariable model were age, sex, socioeconomic position (occupation, education, household income), smoking [35, 36], alcohol use [36, 37], number of comorbidities [38, 39] and self-rated health [40, 41]. Occupation referred to the occupation that participants had for the longest period in their life. The presence of comorbidities was defined by presence of two or more chronic diseases: diabetes, hypertension, self-reported cancer and self-reported cardiovascular disease. Diabetes was defined by fasting plasma glucose ≥ 7.0 mmol/L or a self-reported medication history, and hypertension was defined by systolic blood pressure ≥ 130 mmHg, diastolic blood pressure ≥ 90 mmHg, and/or taking anti-hypertensive medication [26].

### Statistical analysis

Pearson chi-square test and one-way analysis of variance (ANOVA) were used to compare categorical and continuous variables between groups, respectively. Multivariable linear regression was used to assess the association between hearing loss and GDS-15 scores, reporting adjusted regression coefficient (β) and 95% confidence interval (CI). Logistic regression model was used to assess the association between hearing loss and the presence of depressive symptoms, giving odds ratio (OR) and 95% CI. Mediation analysis was conducted to assess the proportion of the association mediated through social isolation. The “medeff” package in Stata was used to perform mediation analysis. We also performed sensitivity analyses by (1) using different cutoff value of GDS-15 score for the definition of depressive symptoms, and (2) excluding those with hearing aids. Data analysis was done using STATA/SE 15.1.

## Results

Of 10,088 participants recruited from 2006–2008, after excluding 1,126 (11.2%) participants with missing data, 8,962 participants (74.3% women) with complete data on all variables of interest were included in the current analysis.

Table 1 shows that mean age of the participants was 60.2 years, 74.3% were women, and the prevalence of hearing loss was 6.8%. Participants with poor hearing were older, had lower family income, lower education, and higher proportion of having manual occupation, fewer current alcohol users and more current smokers, higher prevalence of poor self-rated health, comorbidities and social isolation (*P* from 0.001 to 0.01). Those with poor hearing also had higher scores of GDS-15 and prevalence of depressive symptoms (both *P* < 0.001) (Table 1).

**Table 1:** Characteristics of participants by hearing status

	**All**	**Hearing status**			***P* value**
		**Good**	**Fair**	**Poor**	
**Number of participants (%)**	8,962	2,256 (25.2)	6,094 (68.0)	612 (6.8)	-
**Age, years, mean (SD)**	60.2 (7.8)	58.3 (7.1)	60.3 (7.6)	65.9 (8.9)	< 0.001
**Age group, years, %**					< 0.001
50–59	54.6	65.6	53.1	28.4	
60–69	30.5	25.0	32.2	33.3	
≥ 70	15.0	9.4	14.7	38.2	
**Sex, %**					< 0.001
Women	74.3	77.1	74.3	63.6	
Men	25.8	22.9	25.8	36.4	
**Occupation, %**					< 0.001
Manual	62.4	58.1	63.1	70.9	
Non-manual	20.8	22.2	20.9	14.9	
Other	16.8	19.7	16.0	14.2	
**Education, %**					< 0.001
≤ Primary	38.1	29.3	39.6	55.2	
Middle school	53.6	61.0	52.5	37.6	
≥ College	8.3	9.8	7.9	7.2	
**Family income, CNY/year, %**					< 0.001
< 10,000	5.5	4.1	5.4	11.0	
10,000–29,999	30.8	30.4	30.7	33.5	
30,000–49,999	25.7	31.7	24.3	17.4	
≥ 50,000	22.0	23.8	22.2	14.2	
Not know	16.1	10.0	17.5	24.0	
**Smoking status, %**					< 0.001
Never	81.8	83.3	82.0	74.4	
Former	7.8	6.8	7.8	11.8	
Current	10.3	9.8	10.2	13.9	
**Alcohol use, %**					0.01
Never	54.0	51.1	54.8	56.5	
Former	5.4	5.3	5.3	6.4	
Current	40.7	43.8	39.9	37.1	
**Self-rated health, %**					< 0.001
Good/ very good	78.5	84.1	77.9	63.9	
Poor /very poor	21.5	15.9	22.1	36.1	
**Comorbidities, %**					0.01
No	88.9	90.3	88.8	85.6	
Yes	11.1	9.7	11.2	14.4	
**Social isolation, %**					< 0.001
No	66.7	72.0	66.1	53.4	
Yes	33.3	28.0	33.9	46.6	
**GDS-15 score, median (IQR)**	2.0 (2.0)	1.0 (3.0)	2.0 (3.0)	2.0 (3.0)	< 0.001
**Depressive symptoms, %**					< 0.001
No	85.3	90.9	84.1	76.0	
Yes	14.7	9.1	15.9	24.0	

*SD* Standard deviation, *CNY* Chinese Yuan, *GDS-15* 15-item Geriatric Depression Scale, *IQR* Interquartile rang

Table 2 shows that after adjusting for age, sex, household income, education, occupation, smoking, alcohol use, self-rated health, comorbidities, compared with participants who had normal hearing, those with fair hearing (β = 0.59, 95% CI: 0.48, 0.69) or poor hearing (β = 0.74, 95% CI: 0.54, 0.94) had higher scores of GDS-15. After similar adjustment, those with poor hearing (OR = 2.13, 95% CI: 1.65–2.74) or fair hearing (OR = 1.68, 95% CI: 1.43–1.99) also showed higher odds of depressive symptoms (Table 2). After additionally adjusting for social isolation, the associations of poor hearing and fair hearing with depressive symptoms attenuated slightly but not substantially, with the β (95% CI) for GDS-15 being 0.69 (0.50–0.89) and 0.58 (0.47–0.68), and the OR (95% CI) for depressive symptoms 2.05 (1.59–2.64) and 1.66 (1.41–1.97), respectively.

**Table 2:** Association of hearing loss with GDS-15 scores and depressive symptoms

Hearing status	Crude model	Model 1	Model 2	Model 3
	**Outcome = GDS scores, β (95% CI)**			
**Good**	Ref (0.00)	Ref (0.00)	Ref (0.00)	Ref (0.00)
**Fair**	0.74 (0.63, 0.85)^***^	0.67 (0.56, 0.78)^***^	0.59 (0.48, 0.69)^***^	0.58 (0.47, 0.68)^***^
**Poor**	1.22 (1.02, 1.42)^***^	1.01 (0.80, 1.21)^***^	0.74 (0.54, 0.94)^***^	0.69 (0.50, 0.89)^***^
	**Outcome = Depressive symptoms, OR (95% CI)**			
**Good**	Ref (1.00)	Ref (1.00)	Ref (1.00)	Ref (1.00)
**Fair**	1.89 (1.61, 2.21)^***^	1.79 (1.52, 2.10)^***^	1.68 (1.43, 1.99)^***^	1.66 (1.41, 1.97)^***^
**Poor**	3.16 (2.50, 4.00)^***^	2.64 (2.07, 3.38)^***^	2.13 (1.65,2.74)^***^	2.05 (1.59, 2.64)^***^

*Ref* Reference, *CI* Confidence interval, *GDS-15* the 15-item Geriatric Depression Scale, *OR* Odds ratio

Model 1: adjusted for age, sex, income, education, occupation, smoking, alcohol use

Model 2: additionally adjusted for self-rated health, comorbidities

Model 3: additionally adjusted for social isolation

^***^P < 0.001

Table 3 shows that the association between poor hearing and depressive symptoms did not vary by sex, age group or education (*P* values for interaction from 0.38 to 0.97). Mediation analysis showed that the proportion of the association mediated through social isolation was 13% (95% CI: 0.10, 0.19) and the results remained after adjusting for potential confounders (9%, 95% CI: 0.06, 0.22) (Table not shown).

**Table 3:** Association of hearing loss with depressive symptoms by sex, age groups and education

**Subgroup**		**Hearing status**	**OR (95% CI)**^ab^	**P for interaction**
**Sex**	Men	Fair	1.66 (1.18, 2.35)^**^	0.38
		Poor	2.11 (1.31, 3.42)^**^	
	Women	Fair	1.70 (1.41, 2.05)^***^	
		Poor	2.17 (1.61, 2.93)^***^	
**Age, years**	50–59	Fair	1.60 (1.30, 1.96)^***^	0.97
		Poor	2.11 (1.39, 3.19)^***^	
	60–69	Fair	2.11 (1.49, 3.00)^***^	
		Poor	2.64 (1.63, 4.27)^***^	
	≥ 70	Fair	1.46 (0.91, 2.32)	
		Poor	1.93 (1.12, 3.31)^*^	
**Education**	≤ primary	Fair	1.51 (0.89, 2.56)	0.95
		Poor	2.03 (1.05, 3.92)^*^	
	middle school	Fair	1.72 (1.26, 2.34)^**^	
		Poor	2.27 (1.48, 3.48)^***^	
	≥ college	Fair	1.61 (1.20, 2.15)^**^	
		Poor	2.36 (1.44, 3.85)^**^	

*Ref* Reference, *OR* Odds ratio, *CI* Confidence interval

^a^The reference group was good hearing

^b^Adjusted for age, sex, income, education, occupation, smoking, alcohol use, self-rated health and comorbidities

^*^P < 0.05

^**^P < 0.01

^***^P < 0.001

Sensitivity analyses using GDS-15 score of 8 or more as the cut-off point to define the presence of depressive symptoms showed similar results. Poor hearing was significantly associated with higher odds of depressive symptoms (adjusted OR = 1.78, 95% CI: 1.16–2.73) (Supplementary table 3). After excluding those who used hearing aids, the association of poor hearing with depressive symptoms appeared to be more pronounced (OR = 1.88, 95% CI: 1.22–2.89) (Supplementary table 4).

## Discussion

Our study showed that hearing loss was independently associated with a higher risk of depressive symptoms, and the results did not vary by age, sex or education. The results attenuated slightly after adjustment for social isolation, indicating that other pathways might be involved. We also found that about 9% of the association could be explained by social isolation, highlighting the potential intervention approaches to prevent depression.

Our results that social isolation did not fully explain the association between poor hearing and depressive symptoms suggest further investigation to shed light on the exact mechanisms. We found a previous study showing the association between hearing loss and depressive symptoms attenuated and became non-significant after adjustment for social contact [25], while another two studies showing that the association remained significant after adjustment for social contact [42, 43]. Our results of subgroup analysis showed that the association was the strongest for those aged 60 to 69 years, but attenuated to the null in those aged over 70 years. As older people may have adapted to changes in their hearing status, with the communication skills changed or improved, the detrimental effects of hearing impairment on mental health maybe attenuated and the impact on social isolation might not be substantial [43]. Additionally, older people could use hearing aids to improve their hearing status and thus may alleviate the negative impacts induced by hearing loss [43]. However, our sensitivity showed similar results after excluding the participants using hearing aids. Future randomized controlled trials to confirm the effect of hearing aids use on improving mental health in older adults are warranted.

Neuropsychological mechanisms might underlie the association between hearing loss and depressive symptoms. For example, a decline in grey matter volume in temporal gyri, frontal gyri, primary auditory cortex and hypothalamus was found in neuroimaging studies on hearing loss [44–47]. Similar changes in cortical and subcortical was also observed in those with depression [48–50]. Besides, a study examining the effect of hearing loss on auditory and emotional networks found that amygdala and parahippocampal of participants with hearing loss showed decreased responses to emotional sounds [51]. However, the exact mechanisms are unclear. Further studies are needed to clarify the underlying pathophysiology of the association between hearing loss and depression.

Our findings further highlight the effect of hearing loss on depressive symptoms in older people as well as the pathway mediated through social isolation, which deserves further attention of clinician and audiologists. Audiologists or social healthcare workers are encouraged to provide emotional support and address the psychosocial needs for individuals with hearing loss, which might be helpful to improve mental health [52, 53]. However, despite the existing need by individuals with hearing loss, to date healthcare services for hearing-impaired individuals are mainly focused on treatment [54] and psychosocial support is rarely provided to them for coping with the social difficulties related to hearing loss [55]. This calls for effective interventions to tackle the issues related to increasing prevalence and detrimental impact of both social isolation and hearing loss.

Strengthens of our study included the large number of participants and a quantitative mediation analysis on social isolation. However, our study also had some limitations. First, causal inference could not be confirmed in the current study using cross-sectional analysis. Second, hearing status was classified by self-report. Further studies with hearing status measured by pure-tone audiometry are warranted [56, 57]. Third, we could not assess the association of hearing aids use with depressive symptoms because of the limited number of participants using hearing aids (*n* = 20). Fourth, our participants might not be fully representative to the general population due to the oversampling of women. Nevertheless, we adjusted for sex in the regression models as well as tested the interaction between hearing loss and sex, and we found no evidence that the associations varied by sex. Thus the unbalanced sex ratio might not be a major concern. Fifth, as our data were collected by interview, those with hearing difficulty might not be able to answer the questions correctly due to their poor hearing. However, as the interviews were conducted one-on-one and face-to-face by well-trained nurses using a computer-assisted questionnaire, the impact of hearing difficulty on hearing and answering the questions correctly was minimized.

In conclusion, our study showed that poor hearing was associated with a higher risk of depressive symptoms, and the association could not be fully explained by social isolation. Further investigation to shed light on the exact mechanisms behind this phenomenon is warranted. Health professionals and clinical practitioners are recommended to increase their awareness and understanding of depressive symptoms related to hearing status.

## Supplementary Information

**Additional file 1:** **Supplementary Table 1. **The four types of social isolation question and thescoring criteria for the composite social isolation score. **Supplementary Table2.** The Chinese version of four types of social isolation question and thescoring criteria for the composite social isolation score. **Supplementary Table3.** Associationof hearing loss with depressive symptoms (GDS-15 score ≥8). **Supplementary Table4.** Association of hearingloss with depressive symptoms after excluding participants with hearing aids (*n*=20).

## Data Availability

Ethical approval in place allows us to share data on requests. Please directly send such requests to the Guangzhou Biobank Cohort Study Data Access Committee (gbcsdata@hku.hk).

## Abbreviations

GDS-15: The 15-item Geriatric Depression Scale
CI: Confidence interval
OR: Odds ratio
GBCS: Guangzhou Biobank Cohort Study
GHHARE: Guangzhou Health and Happiness Association for the Respectable Elders
UK: United Kingdom
RMB: Renminbi
USD: United States Dollar
SNI: Social Network Index
ANOVA: Analysis of variance
SD: Standard deviation
CNY: Chinese Yuan
IQR: Interquartile range
Ref: Reference
