# Supplementary material for: Hearing loss and depressive symptoms in older Chinese: whether social isolation plays a role
Source: BMC Geriatr. 2022 Jul 26;22:620. doi: 10.1186/s12877-022-03311-0 (PMC9316428; doi:10.1186/s12877-022-03311-0)
Supplement: Supplementary file 1 — Additional file 1: Supplementary Table 1. The four types of social isolation question and thescoring criteria for the composite social isolation score. Supplementary Table2. The Chinese version of four types of social isolation question and thescoring criteria for the composite social isolation score. Supplementary Table3. Associationof hearing loss with depressive symptoms (GDS-15 score ≥8). Supplementary Table4. Association of hearingloss with depressive symptoms after excluding participants with hearing aids (n=20). [file 12877_2022_3311_MOESM1_ESM.pdf]

Supplementary table 1. The four types of social isolation question and the scoring criteria for the composite social isolation score.

Items^	Questions	Answers	Scoring		
			**Score of 0 if:**	**Score of 1 if:**	**Score of 2 if**
Face-to-face contact with co-inhabitants	How many people do you spend time with on a regular basis (at least once a month; do not include phone/mail conversations; excluding the workplace; including family)?	"≥3people", "< 3 people" and "Live alone	≥ 3 people	< 3 people	Live alone
Face-to-face contact with non-co-inhabitants	How often do you see any of your friends or relatives (do not include phone/mail conversations; excluding those who lived together)?	"≥1 time/month", "< 1 time/month" and "No such contact"	≥ 1 time /month	< 1 time/month	No such contact
Non-face-to-face contact (by telephone/mail)	Do you have any relatives or friends you don't often see but keep in contact with by phone or letter? How often do you contact them?	"≥1 time/month", "< 1 time/month", and "No such contact"	≥ 1 time /month	< 1 time/month	No such contact
Club/organization contact	How often do you join in activities of the GHHARE or other organizations?	"≥1 time/month" and "< 1 time/month"	≥ 1 time /month	< 1 time/month	NA
Composite score: sum of scores			0~7		

Supplementary table 2. The Chinese version of four types of social isolation question and the scoring criteria for the composite social isolation score.

类型	问题	回答	得分		
			0 分:	1 分:	2 分
与同住者面对面接触	与您经常交往的人有多少个?(至少每月一次;不包括电话交谈；不包括工作场所，但包括家人)	“不少于 3 人”，“少于 3 人” 和 “独居”	不少于 3 人	少于 3 人	独居
与非共同居住者面对面接触	与您的朋友、亲戚见面的频率（不包括与您同住的人）	“至少每月一次”，“少于每月一次” 和 ″没有接触″	至少每月一次	少于每月一次	没有接触
非面对面接触（通过电话/邮件）	您是否有一些朋友或亲戚，虽然没经常见面，但通过电话和信件保持联系?如果是,您与他们联系的频率是？	“至少每月一次”，“少于每月一次” 和 ″没有接触″	至少每月一次	少于每月一次	没有接触
俱乐部/组织的接触	您是否有加入团体或协会? 如果有,您参加他们活动的频率?	“至少每月一次”和“少于每月一次”	至少每月一次	少于每月一次	
总分：				0~7	

Supplementary table 3. Association of hearing loss with depressive symptoms (GDS-15 score ≥8)

Hearing status	Depressive symptoms (GDS-15 scores≥8), OR (95% CI)				
	**Number (% cases)**	**Crude model**	**Model 1**	**Model 2**	**Model 3**
**Good**	2256 (2.53)	Ref (1.00)	Ref (1.00)	Ref (1.00)	Ref (1.00)
**Fair**	6094 (4.64)	1.88 (1.41, 2.51)***	1.74 (1.30, 2.34)***	1.57 (1.17, 2.11)**	1.54 (1.15, 2.08)**
**Poor**	612 (7.35)	3.06 (2.05, 4.58)***	2.40 (1.58, 3.65)***	1.78 (1.16, 2.73)**	1.70 (1.10, 2.62)*

Ref=reference; OR=odds ratio; CI=confidence interval; GDS-15=the 15-item Geriatric Depression Scale

Model 1: adjusted for age, sex, income, education, occupation, smoking, alcohol use

Model 2: additionally adjusted for self-rated health, comorbidities

Model 3: additionally adjusted for social isolation

**P<0.05

**P<0.01

***P<0.001

Supplementary table 4. Association of hearing loss with depressive symptoms after excluding participants with hearing aids (n=20)

Hearing status	Depressive symptoms, OR (95% CI)		
	**Crude model**	**Model 1**	**Model 2**
**Good**	Ref (1.00)	Ref (1.00)	Ref (1.00)
**Fair**	1.90 (1.42, 2.55)***	1.77 (1.32, 2.38)***	1.60 (1.19, 2.16)**
**Poor**	3.21 (2.14, 4.80)***	2.52 (1.65, 3.84)***	1.88 (1.22, 2.89)**

Ref=reference; OR=odds ratio; CI=confidence interval

Model 1: adjusted for age, sex, income, education, occupation, smoking, alcohol use

Model 2: additionally adjusted for self-rated health, comorbidities

**P<0.01

***P<0.001
